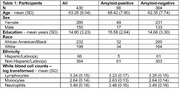# White blood cell types and tau deposition in the medial temporal lobe of cognitively normal older adults

**DOI:** 10.1002/alz.093280

**Published:** 2025-01-03

**Authors:** Meredith N Braskie, Noelle Lee, Marylan Davison, Victoria R Tennant, Koral V Wheeler, Meral A Tubi, Jamie Terner, Maxwell W Hand, Arthur W. Toga, Sid E. O'Bryant, Kristine Yaffe

**Affiliations:** ^1^ Stevens Neuroimaging and Informatics Institute, Los Angeles, CA USA; ^2^ Imaging Genetics Center, Mark and Mary Stevens Neuroimaging and Informatics Institute, Keck School of Medicine, University of Southern California, Marina del Rey, CA USA; ^3^ Stevens Neuroimaging and Informatics Institute, Marina del Rey, CA USA; ^4^ Mark and Mary Stevens Neuroimaging and Informatics Institute, Keck School of Medicine, University of Southern California, Los Angeles, CA USA; ^5^ Stevens Neuroimaging and Informatics Institute, University of Southern California, Los Angeles, CA USA; ^6^ Laboratory of Neuro Imaging, USC Stevens Neuroimaging and Informatics Institute, Keck School of Medicine, University of Southern California, Los Angeles, CA USA; ^7^ University of North Texas Health Science Center, Fort Worth, TX USA; ^8^ University of California, San Francisco, Weill Institute for Neurosciences, San Francisco, CA USA

## Abstract

**Background:**

Alzheimer’s disease (AD) and other dementia risk may be influenced by the immune function and associated with several white blood cell type counts. In cognitively normal Black, Hispanic, and non‐Hispanic white older adults we related three white blood cell types previously associated with AD risk to tau positron emission tomography (PET) values in the medial temporal lobe (MTL), where tau accumulates early. We assessed whether amyloid positivity moderated this relationship.

**Method:**

We evaluated 430 cognitively normal older adults (Table 1) from the Health & Aging Brain Study‐Health Disparities (HABS‐HD) study who had: 1) MTL tau PET values 2) counts for three types of white blood cells: absolute monocytes, lymphocytes, and neutrophils and 3) global brain amyloid values. Tau was assessed using [18F]PI‐2620 PET standardized uptake value ratios (SUVR; inferior cerebellar gray matter reference region). The MTL region included FreeSurfer‐derived hippocampus, amygdala, and entorhinal cortex. Global amyloid ([18F]Florbetaben PET) was calculated as mean SUVR in frontal, cingulate, lateral parietal, and lateral temporal cortex using a whole cerebellar reference region. Multiple linear regression related log‐transformed absolute counts of white blood cell types to MTL tau, covarying for age, sex, education, ethnicity (Hispanic or non‐Hispanic) and race (Black or white) with white blood cell type by amyloid positivity (global amyloid SUVR >1.08) interactions modeled on MTL tau.

**Result:**

Amyloid‐positivity interacted with lymphocytes on MTL tau (p = 0.02); in amyloid‐positive participants there was a stronger relationship between higher lymphocytes and more MTL tau (β = 0.21) than in amyloid‐negative participants (b = 0.02). There was a trend level interaction between amyloid positivity and monocytes on MTL tau (p = 0.06); in amyloid‐positive participants there was a stronger relationship between lower monocytes and more MTL tau (β = ‐0.23) than in amyloid‐negative participants (β = ‐0.10). Older age (β = 0.40, p<0.001), female sex (β = 0.11, p = 0.02), Black race (β = 0.20, p<0.001), and Hispanic ethnicity (β = 0.22, p<0.001) also were associated with greater MTL tau.

**Conclusion:**

Higher lymphocyte and lower monocyte values were associated with more MTL tau, particularly in amyloid‐positive older adults suggesting a complex relationship between the peripheral immune system and early AD neuropathology.